# Political commitment for vulnerable populations during donor transition

**DOI:** 10.2471/BLT.16.179861

**Published:** 2017-02-01

**Authors:** Daniela C Rodríguez, Alan Whiteside, Sara Bennett

**Affiliations:** a Department of International Health, Johns Hopkins Bloomberg School of Public Health, 615 North Wolfe Street, Baltimore, MD 21205, United States of America.; b Centre for International Governance Innovation, Balsillie School of International Affairs, Waterloo, Canada.

## Abstract

The responsibilities for the programmatic, technical and financial support of health programmes are increasingly being passed from external donors to governments. Programmes for family planning, human immunodeficiency virus, immunization, malaria and tuberculosis have already faced such donor transition, which is a difficult and often political process. Wherever programmes and services aimed at vulnerable populations are primarily supported by donors, the post-transition future is uncertain. Overreliance on donor support is often a reflection of limited domestic political commitment. Limited commitment, which is frequently expressed as the persecution of vulnerable groups, poses a risk to individuals as well as to the effectiveness and sustainability of health programmes. We argue that, for reasons linked to human rights, the social contract and the cost–effectiveness of health promotion, prevention and treatment programmes, it is critical that governments sustain health services for vulnerable populations during and after donor transition. Although civil society organizations could help by engaging with government stakeholders, pushing to change social norms and supporting mechanisms that demand accountability, they may be constrained by economic, political and social factors. Vulnerable populations need to be actively involved in the planning and implementation of donor transition – to ensure that their voice and needs are taken into account and to establish a platform that improves visibility and accountability. As transitions spread across all aspects of global health, transparent conversations about the building and sustainment of political commitment for health services for vulnerable populations become a critical human rights issue.

## Résumé

Aujourd'hui, les responsabilités pour le soutien programmatique, technique et financier des programmes de santé sont de plus en plus souvent transférées de donateurs extérieurs aux gouvernements. Les programmes liés à la planification familiale, au virus de l'immunodéficience humaine, aux vaccinations, au paludisme et à la tuberculose ont déjà amorcé ce type de transition, qui constitue un processus difficile et souvent politique. Partout où des programmes et services ciblant des populations vulnérables sont principalement financés par des donateurs, le futur post-transition est incertain. La sur-dépendance à des donations externes traduit souvent un engagement politique national limité. Or, un engagement limité (qui se manifeste fréquemment par la persécution de groupes vulnérables) crée un risque pour les individus mais aussi pour l'efficacité et la pérennisation des programmes de santé. Selon nous, pour des raisons liées aux droits de l'homme, au contrat social et à la rentabilisation des programmes de promotion, prévention et traitement de santé, il est crucial que les gouvernements soutiennent les services de santé destinés aux populations vulnérables, pendant et après cette transition qui affecte les sources de financement. Même si les organisations de la société civile peuvent être utiles, en s'engageant auprès des acteurs gouvernementaux et en faisant pression pour changer les normes sociales et promouvoir des mécanismes de responsabilisation, elles sont parfois entravées dans leur action par des facteurs économiques, politiques et sociaux. Les populations vulnérables doivent être activement impliquées dans la planification et la mise en œuvre de la transition des sources de financement pour que leurs voix et leurs besoins soient pris en compte et pour créer une plate-forme qui améliore la visibilité et la responsabilisation. À l'heure où ce type de transition s'étend à tous les domaines sanitaires mondiaux, la tenue de débats transparents sur la création et le maintien de l'engagement politique en faveur des services de santé destinés aux populations vulnérables devient un enjeu essentiel en termes de respect des droits de l'homme.

## Resumen

Cada vez más, las responsabilidades del apoyo programático, técnico y financiero de programas sanitarios se pasan de los donantes externos a los gobiernos. Los programas de planificación familiar, del virus de la inmunodeficiencia humana, de la inmunización, de la malaria y de la tuberculosis ya han experimentado dicha transición de donantes; se trata de un proceso complicado y, a menudo, político. Allí donde los programas y servicios diseñados para poblaciones vulnerables reciben, principalmente, el apoyo de los donantes, el futuro después de la transición es incierto. El exceso de confianza en el apoyo de los donantes suele ser un reflejo del escaso compromiso político nacional. Un compromiso escaso, que suele expresarse como la persecución de grupos vulnerables, supone un riesgo para los individuos, así como para la eficacia y sostenibilidad de los programas sanitarios. El argumento ofrecido es que, por razones vinculadas a los derechos humanos, el contrato social y la rentabilidad de los programas de fomento sanitario, prevención y tratamiento, es fundamental que los gobiernos mantengan los servicios sanitarios para poblaciones vulnerables durante y después de la transición de donantes. A pesar de que organizaciones de la sociedad civil pueden ayudar colaborando con los participantes gubernamentales, fomentando normas de cambio social y apoyando mecanismos de rendición de cuentas, pueden verse limitadas por factores económicos, políticos y sociales. Las poblaciones vulnerables necesitan involucrarse de forma activa en la planificación y la implementación de la transición de donantes a fin de garantizar que su voz y sus necesidades se tengan en cuenta y para establecer una plataforma que mejore su visibilidad y su responsabilidad. Conforme las transiciones se van ampliando en todos los aspectos de la sanidad global, las conversaciones transparentes sobre la construcción y el mantenimiento de un compromiso político ante los servicios sanitarios para poblaciones vulnerables se han convertido en un asunto de derechos humanos fundamental.

## ملخص

يتزايد التوجه الرامي إلى نقل مسؤوليات الدعم الهيكلي والتقني والمالي لبرامج الصحة من على عاتق الجهات الخارجية المانحة إلى الحكومات. وقد تعرضت برامج تنظيم الأسرة ومكافحة فيروس نقص المناعة المكتسب والملاريا والسل بالفعل إلى هذه الحالات من تغيير الجهات المانحة، والتي غالبًا ما تمثل عملية سياسية وعسيرة. فكلما اعتمدت البرامج والخدمات التي تهدف إلى مساعدة الشرائح السكانية الهشة بشكل أساسي على مساعدات الجهات الخارجية المانحة، بدا مستقبل ما بعد سياسة تغير الجهات المانحة غير واضح. وغالبًا ما يعكس الاعتماد المفرط على الدعم المقدم من الجهات المانحة التزامًا سياسيًا محدودًا داخل البلاد. وتشكل محدودية الالتزام السياسي الذي يتمثل دائمًا في اضطهاد الشرائح السكانية الهشة خطرًا على أفراد المجتمع ومدي فاعلية برامج الصحة واستدامتها. وحرصًا من جانبنا على أهمية العقد الاجتماعي وتعزيز النظم الصحية بشكل فعال من حيث التكلفة وبرامج العلاج والوقاية لأسباب تتعلق بحقوق الانسان، فإننا ندعو إلى ضرورة قيام الحكومات بدعم الخدمات الصحية للشرائح السكانية الهشة خلال وبعد فترة تغير وتحول الجهات المانحة. وبالرغم من أن منظمات المجتمع المدني قد تقدم يد العون من خلال المشاركة مع الجهات الحكومية المعنية، والدفع في اتجاه تغيير المعايير الاجتماعية، ودعم الآليات التي تطالب بالمساءلة، إلا أنها قد تتقيد بالعوامل الاجتماعية والسياسية والاقتصادية. ويلزم إشراك الشرائح السكانية الهشة في عملية تخطيط وتنفيذ سياسة تغير الجهات المانحة لضمان أخذ أصواتهم واحتياجاتهم بعين الاعتبار وإنشاء آلية لتعزيز المساءلة ووضوح الرؤية. وبينما انتشرت سياسة تحول الجهات المانحة بشكل واسع على جميع المستويات الصحية العالمية، أصبحت المحادثات الشفافة حول تعمير ودعم الالتزام السياسي لتقديم خدمات الصحية للشرائح السكانية الهشة ضرورة وحقًا إنسانيًا.

## 摘要

为健康计划提供程序、技术及财政支持的责任正逐渐从外部捐助者转移给政府。 计划生育、人类免疫缺陷病毒、免疫接种、疟疾以及结核病计划已面临这一捐助者过渡问题，这是一个艰难的过程，通常为政治过程。 无论针对弱势群体的计划与服务在何处受到捐助者的重点支持，实现后过渡的前景都并不明朗。 过度依赖捐助者支持往往反映出国内政治承诺的局限性。 有限承诺通常表现为对弱势群体的迫害，为个人以及健康计划的有效性和可持续性构成了风险。 我们认为，由于与人权以及健康促进、预防和治疗计划的社会契约性与经济效益相关的原因，政府在捐助者过渡期以及过渡期之后继续为弱势群体提供健康服务很关键。 尽管民间社会团体能通过与利益相关者合作、推动改变社会规范并支持责任追究机制提供帮助，但是他们也许会受到经济、政治和社会因素的约束。 弱势群体需要积极参与捐助者过渡的规划和实施，以确保其心声和需求得到重视，并创建一个提高知名度并改善问责制的平台。 由于过渡扩散到全球健康的各个方面，有关建立和维护对弱势群体健康服务的政治承诺的坦诚对话成为重要的人权问题。

## Резюме

Ответственность за разработку, техническую и финансовую поддержку программ здравоохранения все больше перекладывается с внешних доноров на правительства. Программы, связанные с планированием семьи, вирусом иммунодефицита человека, иммунизацией, малярией и туберкулезом, уже столкнулись с подобной передачей ответственности доноров, что является сложным и часто политическим процессом. Во всех случаях будущее программ и служб, нацеленных на работу с уязвимыми группами населения, которые изначально поддерживались донорами, после переноса ответственности становится неопределенным. Излишняя надежда на донорскую помощь часто свидетельствует об ограниченности обязательств внутренней политики, которая нередко выражается в виде дискриминации уязвимых групп населения и представляет угрозу как для отдельных людей, так и для эффективности и устойчивости программ здравоохранения. Авторы уверены, что по причинам, имеющим отношение к правам человека, общественному договору и экономической эффективности программ по укреплению здоровья, профилактике и лечению, важно, чтобы правительства поддерживали существование служб здравоохранения для уязвимых групп населения во время и после передачи ответственности доноров. Хотя общественные организации могут помочь путем привлечения заинтересованных лиц из правительственных кругов, агитации за изменение социальных норм и поддержки механизмов, требующих подотчетности, они могут оказаться под давлением экономических, политических и социальных факторов. Уязвимые группы населения должны принимать активное участие в планировании и осуществлении передачи ответственности доноров, чтобы их мнение и потребности были приняты во внимание и чтобы заложить основы для развития прозрачности и подотчетности. Передача ответственности касается всех аспектов мирового здравоохранения, и открытый диалог по принятию и выполнению политических обязательств в отношении служб здравоохранения для уязвимых групп населения становится решающим вопросом соблюдения прав человека.

## Introduction

In a so-called donor graduation or transition, a donor transfers the responsibility for the financial, managerial and/or technical support of a programme to one or more local stakeholders – usually government. Such transitions are becoming more common as donor priorities shift, economic growth and fiscal capacity in low- and middle-income countries increase and pressures on donor budgets rise.^1^ Transitions can be planned – e.g. triggered by the country meeting a certain threshold – or they can be sudden and driven more by political priorities. The United States Agency for International Development’s transition of family planning programmes in Latin America in the 1990s and 2000s,^2^ and the more recent implementation of new eligibility or graduation policies by global health initiatives – e.g. Gavi the Vaccine Alliance and the Global Fund to Fight AIDS, Tuberculosis and Malaria – are examples of triggered donor transition.^3^^,^^4^

The Global Fund’s eligibility criteria – most recently amended in 2016 to include income, disease burden and G20 membership – are used to determine if a country could apply for funds, whether funding should be restricted to work with vulnerable populations and the level of co-financing required.^4^ Gavi primarily uses a country’s gross national income per capita to determine graduation status but bases its support of a new vaccine’s introduction on the country’s coverage with three doses of diphtheria–tetanus–pertussis vaccine.^3^ Both Gavi and the Global Fund use graduation eligibility criteria that focus on national-level indicators and obscure inequalities within a country. The United States President’s Emergency Plan for AIDS Relief (PEPFAR) is less explicit but promotes transition in countries that have low or concentrated epidemics and focuses its investments in generalized epidemics on those geographical areas that have the greatest needs for treatment and prevention.^5^

Regardless of the reasons underlying a donor transition, the process can be highly political. The donors and local stakeholders may disagree, at least initially, about what to expect and how to prepare. Of particular concern are donor-supported programmes for vulnerable, or so-called key, populations. The Global Fund defines key populations as groups of individuals who: (i) have an elevated risk of, vulnerability to, and/or burden of disease because of biological, socioeconomic and/or structural factors; (ii) have substantially poorer access to the relevant services than the rest of the population and require strategic efforts and investments to reach them; and (iii) face criminalization, economic and social marginalization, frequent human rights violations and/or systematic disenfranchisement that increases their vulnerability.^6^

In the field of human immunodeficiency virus (HIV), groups such as female sex workers, men who have sex with men, people who inject drugs and transgender people are considered key populations. For tuberculosis, the key populations include miners, people who inject drugs and prisoners.^7^ The Global Fund’s formal definition of a key population is limited to the Global Fund’s targets of HIV, malaria and tuberculosis. However, we believe that the definition could and should be applied more broadly because it addresses issues of access, equity, human rights and marginalization, which are critical contributors to vulnerability for many health problems. Islamic communities in northern Nigeria, for example, could be viewed as key populations for immunization services because: (i) their low rates of immunization mean that they are at increased risk of contracting vaccine-preventable diseases; (ii) a decay in the regional health service infrastructure has led to low accessibility to immunization services; and (iii) there are systematic issues of confusion, fear and a general lack of trust in government services.^8^

Below, we make the case for the continuation and promotion of key-population-specific services after a donor transition. We argue that key populations often lack political commitment from their governments and, in consequence, their needs may easily be deprioritized after donor transition. We also explore the complex role that external partners can – and do – play in donor transition and the need for greater future transparency and coordination in services targeted at key populations.

## Key-population-specific services

### Justification

A government’s obligation to provide services to different segments of a national population stems primarily from notions of human rights, the broader social contract and the importance of universality. A fundamental component of a government’s authority over its citizens arises from the government’s commitment to protect its citizens’ rights. The right to health is frequently enshrined in a country’s constitution or national standards. Global agendas, such as the sustainable development goals and universal health coverage – emphasize the need for governments to ensure that all of the population’s health needs are met. The agendas also underline the common view that individuals and communities have an inherent value that justifies the provision of services to them. Although governments may claim to be committed to human rights and the protection of even the most vulnerable of their citizens, many of them fail to provide adequate health services to their key populations – because of deliberate policies or passive neglect.^9^

Health services for key populations may require targeted, additional investments. Unfortunately, the principles of human rights and universality usually have to be balanced against those of economics. Policy-makers are often forced to decide whether to allocate limited funds to mainstream service provision – which may benefit the general population but not reach key populations – or to special services targeted at key populations. For example, spending on HIV at the expense of other health areas raises important questions about what to do when resources cannot be reallocated without making anyone worse off, i.e. Pareto efficiency. Such targeted provision might be hard to justify for a government when faced with an obligation to meet the needs of multiple communities.^10^ The equity–efficiency trade-off in health care has been widely debated,^11^^,^^12^ but may be misleading when key populations are being considered. The epidemiological importance of the key populations may be so high that investment in the provision of services for them may serve both the goals of equity and efficiency. For HIV and tuberculosis, for example, the failure to provide key-population-specific prevention services may lead to much greater funding needs in the long term for treatment because the key populations are often the drivers of the spread of infection. In 2014, for example, it was estimated that, compared with other adults, people who inject drugs were 28 times more likely to be infected with HIV.^13^

Therefore, there are strong rights-based arguments and often strong economic and epidemiological arguments for ensuring the provision of key-population-specific services. However, marginalization, stigma and discrimination often weaken the political commitment to such provision and may mean that the concerns of the key populations never even reach the political agenda because of their reduced voice and visibility. This is particularly problematic when the key populations do not have an organized constituency and face hostility – including criminalization and harassment – from their own governments. Several organizations have already warned about the deleterious consequences of donor transition for programmes serving key populations.^1^^,^^14^^–^^16^

### Need for political commitment

Key populations are usually underserved and marginalized. The health services for such populations, where available, are often supported, directly or indirectly, by external donors because the relevant government stakeholders lack the necessary commitment. This is the scenario faced by PEPFAR, in its support of HIV services in Central America, the Dominican Republic and Ukraine,^17^^–^^20^ and by the Global Fund, through its policy to allow middle-income countries with low disease prevalence to remain eligible for key-population-specific support.^4^ Donor transition may expose a government’s reticence to support services targeted at key populations and reveal how external partners had been allowed to fill the gaps. As donor support for programmes lacking political commitment ends, there is considerable risk that any health gains already made will be lost and that the corresponding areas of concern will re-emerge or worsen. In Romania, where Global Fund support for HIV services ended in 2010, the prevalence of HIV infection among people who inject drugs rose from less than 2% in 2006 to 53% in 2012.^1^^,^^21^ Other donor transitions in the field of HIV appear to have led to drops in care and testing services in Bangladesh, threats to services for sex workers in Botswana, decreases in the quality and coverage of services in China and poor coordination in Guyana.^22^

Political will and commitment – e.g. a government that fully defends and supports a service despite other budgetary, epidemiological and technical priorities – are critical components of the development, delivery and sustainment of the services that improve health outcomes for the marginalized. Even if there is expressed commitment through national policies that include key-population-specific services, the institutional commitment to carry out the policies and/or the budgetary commitment to fund them may be lacking. Although, before the Global Fund’s exit, the Ukrainian Government pledged substantial funding to the HIV response, the actual post-transition budgets for the response were only a third of those pledged.^15^ Similarly, despite its pre-transition assurance to provide such support, the Serbian Government has not assumed funding for prison-based services.^15^

The underlying reasons for resistance to supporting a transitioning programme’s activities may be varied. The health issue addressed by the programme may not be seen as such a high priority by country stakeholders as by external donors. For example, tuberculosis programmes may not be prioritized in a country where child mortality from diarrhoea and pneumonia are seen as greater threats. There also may be technical barriers, e.g. limited government capacity to mobilize and engage with marginalized communities effectively and the need for specific recruitment and retention policies for the health workers serving rural and hard-to-reach populations. However, resistance may also be linked to opposition to the services themselves, e.g. specific family planning methods, or to the key populations at which they are targeted, e.g. ethnic minorities or transgender individuals. In such cases, the response is more complex and requires the building and sustainment of political commitment.

### Sustaining political commitment

Most attempts at building political commitment are targeted at national governments because the budgets, laws, policies and regulations that can sustain a health programme in the long term often flow from governments. However, even when it appears that the political commitment established in a national government is sufficient to sustain a programme, the commitment may still evaporate with elections and a new leadership or simply because of shifting priorities and policy concerns. The decentralization of Mexico’s health system in the 1990s, which devolved more decision-making power to state-level officials, coincided with the graduation of family planning in Mexico in the late 1990s. After graduation, state-level officials often deprioritized family planning in light of competing and more immediate, short-term investments like new hospitals. Considerable post-graduation advocacy was needed to increase support for family planning programmes.^23^

It is unlikely that political commitment can be effectively built or sustained by focusing on any single type of stakeholder. Rather, there is a need to understand all of the actors involved in the policy space, e.g. advocacy groups, health-care providers, nongovernmental organizations and programme beneficiaries, who need to support the programme and its transition. There is also a need to understand how all of these actors relate to each other.

Sustainable political commitment for key populations may require the changing of social norms and the improvement of accountability, two long-term processes that donors can support but not impose. An evaluation of its Partnership Framework for Central America indicated that PEPFAR had been providing much-needed leadership and political support for activities aimed at key populations, which would otherwise be absent.^24^ In the same evaluation, civil society organizations were found to doubt that, without donor pressure, national leaders would emphasize efforts with key populations. Services for key populations are often the last to be absorbed following donor graduation. In Mexico, for example, the government’s support of family planning programmes for special populations, i.e. indigenous, rural and youth populations, started late during the graduation process; many of the programmes were never institutionalized and were eventually eliminated.^23^

Local civil society organizations can play a role in changing norms and accountability in various ways. First, through advocacy at multiple levels, such as reaching out to districts and municipalities to gain support and demand accountability from policy-makers and implementers at subnational levels who are on the front line of programme implementation. Second, these organizations can establish accountability mechanisms, such as watchdog groups to provide independent observations of national or even regional processes. Although such activities may link to broader civil society efforts to ensure support for key populations, they are especially critical in sustaining a focus on the needs of key populations during and after donor transition.

It is important to remember, though, that the efficacy of civil society is shaped by, and dependent on, the broader legal and human rights environment under which civil society operates. This may sometimes prohibit critical key-population-specific activities and restrict advocacy and organizing efforts more broadly. Further, not all vulnerable populations can be easily organized into clear-cut institutions. This is a particular problem for populations with behaviours that are criminalized, e.g. in most countries, people who inject drugs and sex workers and, in many countries, men who have sex with men. Advocacy for ethnic minorities is often linked to broader political agendas beyond unmet health needs, which can increase marginalization and complicate donor influence.

Critically, given the disproportionate support that external funders may provide for civil society organizations, important questions remain about the best ways to provide financial and technical assistance to civil society that allow for sustained mechanisms for accountability. Options include the provision of donor resources, either directly or via regional mechanisms or international nongovernmental organizations, for the sustainment of local organizations post-transition. Some evaluations of family planning graduations have indicated that external support remained, or would be, essential post-transition. Such support would probably be needed for the Contraceptive Security Committee in Nicaragua^25^ and for nongovernmental organizations to maintain the provision of family planning services to the underserved in Mexico.^23^ Direct government contracts to civil society organizations have had mixed results. While government regulations often limit this option, such contracts have been used in China and Costa Rica to fund civil society organizations after transition. Although in China, the limits on flexibility and autonomy have been problematic.^1^^,^^22^

We need more evidence on what has and has not worked in mobilizing and protecting key populations around the time of donor transition. We need retrospective research on: (i) the changing of social norms, e.g. what worked for family planning; (ii) the role of umbrella networks and regional funding channels in overcoming national-level resistance to key-population-specific support;^15^ and (iii) contractual approaches for the continued support of civil society organizations. We need immediate evaluation and monitoring of ongoing transition-related changes and outcomes. Both prospective research and retrospective evaluations are needed to assess and understand the associated changes to health services over time.

## Engaging key populations in transition

One critical step in preventing key-population-specific services from declining post-transition is to involve key populations in the planning, implementation and monitoring of the transition. An evaluation of the transition of Avahan – an HIV prevention initiative in India established by the Bill & Melinda Gates Foundation and later transferred to the Indian government – indicated that the key populations were not well informed about the transition and did not understand the organizational changes that had taken place as a result of the transition.^26^ However, these populations were very aware of the changes to service delivery and community mobilization that had occurred.^26^

Global health initiatives’ current tools to assess transition readiness address many aspects of preparedness, e.g. financing, governance, organizational capacity and strategic information, but do not explicitly focus on key populations.^27^^–^^30^ However, a new assessment by the Health Policy Project, funded by PEPFAR, focused exclusively on the sustainability of HIV services and activities for key populations.^14^ This is an important first step. Other global health initiatives should also incorporate key-population-specific components in their assessments to permit a better characterization of what will be needed to sustain services for these critical groups post-transition. Likewise, better programme monitoring around key-population services and support could be used to measure country readiness and progress before and during transition.^16^^,^^22^

Both donors and local stakeholders, including civil society organizations, communities and government, should make the substantive participation of key populations in transition planning and implementation a requirement. Where there are organizations that represent key populations, partnership in transition can be sought. Where key populations are particularly hard to reach or engage, extensive consultation and communication with many members of the key populations will be needed. Such community engagement needs to be more than making a favourable impression and the engagement should cover as many key populations as possible.

An effective transition plan will be context-specific and address the multiple programme components that have key-population-specific implications. For example, convenient and confidential testing and treatment for HIV and tuberculosis, and service delivery models for family planning, immunization and malaria designed to reach nomadic or other hard-to-reach populations. Key-population involvement in transition planning cannot only build better relationships and accountability with governments but also identify alternative avenues and strategies for the continued support of key-population-specific services in the post-transition era.

## Conclusion

The global health community must not allow donor transitions to create greater marginalization of key populations. Some governments have allowed donors to support a service that would otherwise go undelivered because of lack of political commitment to serving the key populations involved. Such governments will find it hard, ethically, to justify any unwillingness to maintain services post-transition. Once a population has been receiving services that meet critical needs, it would be morally wrong to reduce or eliminate those services because donor support was ending. There needs to be a greater focus on fundamental human rights and the associated obligation to sustain services for key populations and so help address the broader issues of equity and vulnerability. There needs to be what the Open Society Foundations has called “rights-based transitions”.^15^

With so many donor transitions, ongoing or imminent, donors need to investigate local stakeholders’ plans for supporting key-population-specific services that have been donor-supported to minimize potential long-term injury. Donors also need to engage key populations and the organizations that support them in every phase of the planning and implementation of transitions. Global health initiatives need to give serious attention to addressing the reticence of governments to support key-population-specific activities. They need to consider alternative transition strategies that allow for sustained support for key populations or better coordinated action to encourage tangible changes in government policy. Both of these approaches, which involve ethical and political considerations and the potential for unintended consequences, need to be considered in open dialogues among donors and between donors and countries. We believe that the time to confront these issues is now.
